# Supplementary material for: Content-rich biological network constructed by mining PubMed abstracts
Source: BMC Bioinformatics. 2004 Oct 8;5:147. doi: 10.1186/1471-2105-5-147 (PMC528731; doi:10.1186/1471-2105-5-147)
Supplement: Additional File 2 — The original results of the above study (non-essential files are deleted to keep the file size under the limit set by BMC bioinformatics). [file 1471-2105-5-147-S2.bz2 › chilibotAdditionalFile2/dip40/549ID7910967E10902/html/CD4_IL16.html]

 


 **CD4** and **IL16** 
  
Found 142 abstracts in PubMed, retrieved 40.  
 

 What does Google say? 
 PDF only 
| .edu only 

---

**Interactive relationship** (e.g. stimulation, inhibition, etc)

**Inhibitory relationship**- interleukin IL 16  [ **IL16** ]  can inhibit the activity of  **CD4**  T lymphocytes.  Ref: 12869037 Clin Exp Immunol, 2003
- BACKGROUND IL 16  [ **IL16** ]  has been described as a natural soluble  **CD4**  ligand with immunosuppressive effects in vitro.  Ref: 12569988 Clin Exp Allergy, 2002
- Theophylline 10 3 M and KF19514 10 6 M inhibited  **CD4**  eosinophil migratory responses induced by IL 16  [ **IL16** ] , but 10 6 M dexamethasone did not.  Ref: 11574756 Int Arch Allergy Immunol, 2001
- Agonists selective for H 2 dimaprit, H 3 4 R alpha methylhistamine, and H 4 clobenpropit were capable of inducing the release of bioactive IL 16  [ **IL16** ]  because CD8 cell supernatants induced  **CD4**  cell migration, which was abrogated by an anti IL 16  [ **IL16** ]  antibody.  Ref: 12235264 J Pharmacol Exp Ther, 2002
- The decrease of  **CD4**  T cells in MM may be mediated by activation of CD8 T cells derived cytokine IL 16  [ **IL16** ] .  Ref: 12191564 Leuk Res, 2002
- The resulting cells responded to IL 16  [ **IL16** ]  in an in vitro chemotaxis assay, and this biologic response could be blocked by anti IL 16  [ **IL16** ]  and anti  **CD4**  Abs as well as by a competitive peptide inhibitor corresponding to a sequence in the C terminal domain of IL 16  [ **IL16** ] .  Ref: 11937573 J Immunol, 2002
**Stimulatory relationship**- Therefore, IL 16  [ **IL16** ]  activation of eosinophils is  **CD4**  mediated to elicit the extracellular release of preformed RANTES and eotaxin, which then in an autocrine fashion act on plasma membrane CCR3 receptors to stimulate both enhanced LTC 4 production and the preferential release of IL 4, but not IL 12, from within eosinophils.  Ref: 11971026 J Immunol, 2002
- The grass pollen season further increased IL 16  [ **IL16** ]  expression and also increased the number of  **CD4**  cells in placebo treated, but not in BDP treated, allergic rhinitis patients.  Ref: 11551252 Allergy, 2001
- Recently, epidermal cells in lesional AD were reported to contain increased messenger m RNA levels of IL 16  [ **IL16** ] , a cytokine that induces chemotactic responses in  **CD4**  T cells, monocytes, and eosinophils.  Ref: 11941319 J Allergy Clin Immunol, 2002
- **CD4**  is a recognized IL 16R, and accordingly anti  **CD4**  Fab, soluble  **CD4** , and a  **CD4**  domain 4 based IL 16  [ **IL16** ]  blocking peptide inhibited the actions of IL 16  [ **IL16** ]  on eosinophils.  Ref: 11971026 J Immunol, 2002
- CONCLUSION Our results demonstrate that increased expression of IL 16  [ **IL16** ]  in T cells and mast cells in active Crohn s disease is associated with increased numbers of  **CD4**  lymphocytes.  Ref: 11709514 Gut, 2001
**Neutral relationship**- Interleukin IL 16  [ **IL16** ]  selectively recruits  **CD4**  cells.  Ref: 11551252 Allergy, 2001
- IL 16  [ **IL16** ]  was expressed by  **CD4**  and CD8 positive T cells.  Ref: 11709514 Gut, 2001
- BACKGROUND Interleukin 16  [ **IL16** ]  IL 16  [ **IL16** ]  acts highly chemotactic on  **CD4**  bearing cells.  Ref: 11556864 Arch Otolaryngol Head Neck Surg, 2001
- Interleukin 16  [ **IL16** ]  IL 16  [ **IL16** ]  attracts  **CD4**  cells to sites of inflammation and plays a role in the interaction of dendritic cells, T cells and B cells.  Ref: 11566629 Eur Cytokine Netw< MedlineTA>Eur Cytokine Netw, 2001
- IL 16  [ **IL16** ]  is a  **CD4**  specific ligand, and RANTES is a C C type chemokine that targets monocytes and lymphocytes.  Ref: 12810540 Endocrinology, 2003
- CONCLUSIONS Interleukin 16  [ **IL16** ]  was mainly expressed in a typical  **CD4**  like pattern in human tonsils.  Ref: 11556864 Arch Otolaryngol Head Neck Surg, 2001
- Theophylline and selective PDE IV inhibitor may prevent airway inflammation by downregulating  **CD4**  expression on eosinophils and inhibiting eosinophil migration through  **CD4**  and IL 16  [ **IL16** ]  interaction.  Ref: 11574756 Int Arch Allergy Immunol, 2001
- IL 16  [ **IL16** ]  is a ligand and chemotactic factor for  **CD4**  T cells.  Ref: 12528894 Cell Res, 2002
- In accordance with other data in the medical literature, we conclude that the most important function of IL 16  [ **IL16** ]  is the chemoattraction of  **CD4**  cells.  Ref: 12001788 Allergy Asthma Proc< MedlineTA>Allergy Asthma Proc, 2002
- **CD4**  serves as a coreceptor for HIV and IL 16  [ **IL16** ] .  Ref: 12213222 Exp Cell Res, 2002
- Our data strongly suggest that  **CD4**  lymphocytes constitute the major cellular source for IL 16  [ **IL16** ] .  Ref: 11556864 Arch Otolaryngol Head Neck Surg, 2001
- CONCLUSIONS IL 16  [ **IL16** ]  is involved in the asthmatic process by recruiting  **CD4**  T cells and thus plays an important role in  **CD4**  cell dependent eosinophilic inflammation in asthmatic airways.  Ref: 11775918 Zhonghua Jie He He Hu Xi Za Zhi, 1999
- Here we show that these effects of  **CD4**  ligation were not observed when anti  **CD4**  antibody was replaced with another  **CD4**  ligand, interleukin 16  [ **IL16** ] , or when the anti CD3 and anti  **CD4**  antibodies were spatially separated by immobilization on different beads.  Ref: 12848849 Immunol Cell Biol< MedlineTA>Immunol Cell Biol, 1999
- Interleukin 16  [ **IL16** ]  IL 16  [ **IL16** ]  has been described as a main cytokine involved in  **CD4**  cell recruitment during inflammation.  Ref: 12169178 Allergy, 2002
- It was also found that the percentages of  **CD4**  and EG2 cells were all positively correlated to IL 16  [ **IL16** ]  levels.  Ref: 11775918 Zhonghua Jie He He Hu Xi Za Zhi, 1999
- Circulating levels of interleukin 16  [ **IL16** ]  IL 16  [ **IL16** ]  were measured, in addition to  **CD4**  T cell counts, plasma HIV RNA levels, and the delayed type hypersensitivity DTH response.  Ref: 11527808 Clin Diagn Lab Immunol, 2001
- AIMS Interleukin 16  [ **IL16** ]  IL 16  [ **IL16** ]  has been characterised as a chemokine with selective chemoattraction for  **CD4**  inflammatory T cells.  Ref: 11709514 Gut, 2001
- IL 16  [ **IL16** ]  is a multi functional cytokine that uses  **CD4**  as a receptor to signal diverse biological activities by target cells including T lymphocytes, monocytes and eosinophils.  Ref: 11727516 Expert Opin Biol Ther, 2001
- CONCLUSIONS These data suggest that TNF alpha induced  **CD4**  eosinophils may contribute to eosinophil migratory responses induced by IL 16  [ **IL16** ] .  Ref: 11574756 Int Arch Allergy Immunol, 2001
- Recently the influx of  **CD4**  lymphocytes has been related to the up regulation of IL 16  [ **IL16** ]  in AEDS skin lesions.  Ref: 12169178 Allergy, 2002
- Interleukin IL 16  [ **IL16** ]  has been reported as playing an important role in the accumulation of  **CD4**  cells.  Ref: 11574756 Int Arch Allergy Immunol, 2001
- The first of these, interleukin 16  [ **IL16** ] , is an immunomodulatory cytokine involved in the regulation of  **CD4**  T cells.  Ref: 11967309 J Virol, 2002
- Recently, IL 16  [ **IL16** ]  was also reported to be a potent chemotactic stimulus for  **CD4**  T lymphocytes and eosinophils in asthma and other pulmonary diseases.  Ref: 11509331 Am J Respir Cell Mol Biol, 2001
- Interleukin 16  [ **IL16** ]  IL 16  [ **IL16** ]  is a cytokine which is mainly produced by CD8 T cells and induces chemotaxis of  **CD4**  T cells and monocytes.  Ref: 11999883 Lupus, 2002
- IL 16  [ **IL16** ] , IL 8, IP 10 and RANTES are participated in the accumulation of  **CD4**  T cell.  Ref: 12233067 Nippon Rinsho, 2002
- IL 16  [ **IL16** ]  plays a role in trafficking of several immune cells and may be a major chemotactic signal for  **CD4**  cells.  Ref: 12001788 Allergy Asthma Proc< MedlineTA>Allergy Asthma Proc, 2002
- IL 16  [ **IL16** ]  expression in LCs in patients with AD correlated with the number of infiltrating  **CD4**  cells r =.72, P =.0017 and was completely downregulated parallel to the clinical response of AD lesions to topical treatment with FK506.  Ref: 11941319 J Allergy Clin Immunol, 2002
- Local expression of IL 16  [ **IL16** ]  seems to play a significant role in the initiation and persistence of the inflammatory process in Crohn s disease, presumably by IL 16  [ **IL16** ]  mediated recruitment of  **CD4**  cells, mostly lymphocytes, into the bowel wall.  Ref: 11709514 Gut, 2001

**Non-interactive relationship** (e.g. studied together, co-existance, homology, etc.)

- METHODS We evaluated the effect of natural allergen exposure during a grass pollen season on IL 16  [ **IL16** ]  expression and number of  **CD4**  cells in nasal mucosa.  Ref: 11551252 Allergy, 2001
- Co treatment of peripheral blood mononuclear cells PBMC with IL 16  [ **IL16** ]  plus IL 2 or IL 15 in vitro selectively expands the population of  **CD4**  T cells.  Ref: 11727516 Expert Opin Biol Ther, 2001
- Furthermore, Mo and AM were separately exposed to the three PM size fractions and supernatants assessed for the T helper  **CD4**  lymphocyte chemoattractant interleukin 16  [ **IL16** ]  IL 16  [ **IL16** ] .  Ref: 12746131 J Toxicol Environ Health A, 2003
- Double immunostaining identified  **CD4**  T cells as the major cellular source of IL 16  [ **IL16** ]  expression.  Ref: 11556864 Arch Otolaryngol Head Neck Surg, 2001
- Here, we show that activation of monocyte derived LC like dendritic cells LLDC through engagement of FcepsilonRI induces the expression of IL 16  [ **IL16** ] , a chemoattractant factor for dendritic cells,  **CD4**  T cells, and eosinophils.  Ref: 11714796 J Immunol, 2001
- RESULTS Prior to the pollen season, the expression of IL 16  [ **IL16** ] , but not the number of  **CD4**  cells, was significantly higher in patients with allergic rhinitis than in healthy control subjects.  Ref: 11551252 Allergy, 2001
- We hypothesize that the double immunostained  **CD4**  ir and IL 16  [ **IL16** ]  ir cells represent activated T cells.  Ref: 11556864 Arch Otolaryngol Head Neck Surg, 2001
- Stimulation of  **CD4**  T cells with IL 16  [ **IL16** ]  primes cells to respond to IL 2, by upregulating the expression of IL 2 receptor p75 CD25.  Ref: 11727516 Expert Opin Biol Ther, 2001
- AIMS To examine if IL 16  [ **IL16** ] , a chemoattractant for  **CD4**  cells, is detectable in airway secretions of ventilated newborns.  Ref: 11893439 Early Hum Dev, 2002
- IL 16  [ **IL16** ]  mediated ligation of  **CD4**  expressed on CD8 T cells was found to induce an intracellular signal that directs migration of these cells in vitro.  Ref: 11756173 Blood, 2002
- The pollen season induced change in IL 16  [ **IL16** ]  expression and in  **CD4**  cells was significantly more pronounced in placebo than in BDP treated patients.  Ref: 11551252 Allergy, 2001
- The increased number of  **CD4**  lymphocytes correlated positively with the increased number of IL 16  [ **IL16** ]  positive cells in Crohn s disease.  Ref: 11709514 Gut, 2001
- IL 16  [ **IL16** ]  promotes leukotriene C 4 and IL 4 release from human eosinophils via  **CD4**  and autocrine CCR3 chemokine mediated signaling.  Ref: 11971026 J Immunol, 2002
- ELISA was used to determine the concentrations of IL 16  [ **IL16** ]  in sputum, and  **CD4**  T cells and activated eosinophils EG2 cells in sputum were detected by immunostaining.  Ref: 11775918 Zhonghua Jie He He Hu Xi Za Zhi, 1999
- **CD4**  eosinophil migration in response to IL 16  [ **IL16** ]  was mostly, if not fully, chemokinetic and this migration was significantly inhibited by Fab of anti  **CD4**  monoclonal antibody.  Ref: 11574756 Int Arch Allergy Immunol, 2001
- R5 tropic HIV 1 glycoprotein 120, but not interleukin 16  [ **IL16** ] , the natural agonist, or X4 tropic glycoprotein 120, inhibited MIP 1beta binding to CCR5 in the presence of monomeric and dimeric soluble  **CD4** .  Ref: 12878220 Biochem Biophys Res Commun, 2003
- The modulation of immune cell costimulatory molecules, CD80, CD86, CD40, and HLA DR, and the release of the  **CD4**  T cell chemoattractant IL 16  [ **IL16** ]  were also investigated.  Ref: 12804531 Clin Immunol, 2003
- MIP 1beta induced G protein activation was further increased by simultaneous stimulation of  **CD4**  with its natural agonist, interleukin 16  [ **IL16** ] .  Ref: 12531905 J Biol Chem, 2003
- The amount of infiltrating  **CD4**  cells was determined by immunohistochemistry and correlated with the corresponding IL 16  [ **IL16** ]  cell number by step sections.  Ref: 11709514 Gut, 2001
- As IL 16  [ **IL16** ]  has been shown to regulate  **CD4**  cell recruitment and activation, and is detected early following airway challenge of individuals with asthma, this two step process for IL 16  [ **IL16** ]  production by epithelial cells may represent a rapid response mechanism in the orchestration of allergic airway inflammation.  Ref: 12594062 Am J Respir Cell Mol Biol, 2003
- We reported previously that fibroblasts from patients with GD express IL 16  [ **IL16** ] , a  **CD4**  specific chemoattractant, and RANTES, a C C chemokine, in response to GD specific IgG GD IgG.  Ref: 12794168 J Immunol, 2003
- Although  **CD4**  is not G protein coupled, pertussis toxin inhibited IL 16  [ **IL16** ]  induced eosinophil activation.  Ref: 11971026 J Immunol, 2002
- METHODS We evaluated the effects of theophylline, KF19514 a selective phosphodiesterase PDE IV inhibitor and dexamethasone on  **CD4**  expression on eosinophils and eosinophil migration in response to IL 16  [ **IL16** ] , a natural soluble ligand of the  **CD4**  molecule.  Ref: 11574756 Int Arch Allergy Immunol, 2001
- IL 16  [ **IL16** ]  is a  **CD4**  specific chemoattractant and RANTES is a C C type chemokine.  Ref: 11777993 J Immunol, 2002
- The ability of costimulated CD8 cells to respond to interleukin 16  [ **IL16** ]  IL 16  [ **IL16** ] , a ligand that binds  **CD4**  and induces cellular chemotaxis, was examined.  Ref: 11756173 Blood, 2002
- There was a significant correlation between the change in IL 16  [ **IL16** ]  expression and the number of  **CD4**  cells.  Ref: 11551252 Allergy, 2001
- BACKGROUND It is currently unknown if interleukin IL 16  [ **IL16** ]  exists in the lungs of ventilated infants, and because the predominant cells in the airways of infants with CLD are  **CD4**  macrophages, we hypothesized that IL 16  [ **IL16** ]  plays a role as a pro inflammatory mediator in lung inflammation.  Ref: 11893439 Early Hum Dev, 2002
- Because IL 16  [ **IL16** ]  is a ligand for  **CD4**  and or an undefined  **CD4**  associated protein, the ability of this multifunctional cytokine to regulate the development of human MCs basophils from nongranulated progenitors residing in cord or peripheral blood was evaluated.  Ref: 11937573 J Immunol, 2002
- Relationship between  **CD4**  CD8 T cell ratio and T cell activation in multiple myeloma reference to IL 16  [ **IL16** ] .  Ref: 12191564 Leuk Res, 2002
- In combination with IL 16  [ **IL16** ] , the beneficial effects of IL 2 may be augmented and specifically targeted to  **CD4**  T cells.  Ref: 11727516 Expert Opin Biol Ther, 2001
- Effect of seasonal allergen exposure on mucosal IL 16  [ **IL16** ]  and  **CD4**  cells in patients with allergic rhinitis.  Ref: 11551252 Allergy, 2001
